# Factors influencing cassava farmers’ choice of climate change adaption practices and its effect on cassava productivity in Nigeria

**DOI:** 10.1016/j.heliyon.2023.e14563

**Published:** 2023-03-15

**Authors:** Olabisi Damilola Omodara, Oluwakemi Abosede Ige, Oluwemimo Oluwasola, Akinsola Temitope Oyebanji, Oluwatunmise Oyebisi Afape

**Affiliations:** Department of Agricultural Economics, Obafemi Awolowo University, Ile-Ife, Nigeria

**Keywords:** MVP, GLM, CSA, Multivariate analysis, Discrete choice model, Random utility theory, Rational choice theory, Climate smart agriculture, Eco-friendly farm practices

## Abstract

This study examined the socio-economic factors influencing choice of climate change adaptation practices and the effects of these practices on cassava productivity in Nigeria. Using a multi-stage sampling technique, structured questionnaire was used to survey 100 cassava farmers. The result was analyzed with a multivariate probit and generalized linear regression models. The result showed male dominance (78%) in cassava farming and the mean age of the cassava farmers was 45.46 ± 9.36 years. About 66% of the farmers belonged to cooperative associations and 67% had access to credit facilities. The multivariate model revealed that age of farmers, gender, education qualification, primary occupation, total income, membership of cooperative associations, farming objectives, farming experience, access to extension visit, access to credit, type of land ownership, farm size and climatic conditions significantly influenced choice of climate change adaptation practices among cassava farmers. The generalized linear model identified farming system, multiple crop types/improved crop varieties used, crop diversification, organic manuring, multiple planting dates, use of alternate fallowing, education and credit access to significantly affect cassava productivity. The study concluded that, eco-friendly methods for adapting to climate change increase cassava productivity. Thus, cassava farmers should be trained on the use of best climate change adaptation practices that can boost cassava productivity. In order to practice climate smart farming, it is important to stress the usage of organic manure and alternate fallowing.

## 1 Introduction

The issue of climate change is crucial for the agricultural industry, particularly in poor nations where farming largely depends on climatic factors like rainfall and temperature. International Panel for Climate Change [22] describes climate change as a major change in climate over a long period of time that is brought on by either natural variability or human activity. Crop cultivation is impacted by human activity, which has been the primary driver of climate change over the past 50 years [[6], [23],^39^]. If the negative effects of climate change are not significantly halted by 2050, the phenomenon is predicted to cause a sizable (22%) reduction in the production of food crops worldwide [40]. The likelihood of short-term crop failure and long-term production reductions increases with changes in precipitation patterns [36]. In addition, the increase in temperatures promotes the growth of weeds and pests while reducing the production of valuable crops. A glaring example is the recent displacement of millions of people, livestock, and other animals, as well as the destruction of thousands of farmlands in the south-western Nigeria due to irregular rainfall, flooding, and food shortages.

One of the most important crops grown in Nigeria that is severely impacted by climate change and climate variability is cassava cultivation [21,36]. With an annual production of over 60 million tonnes of cassava tubers, Nigeria is recognized as the world's largest cassava producer [18]. Cassava tuber is crucial to the development of Nigeria's food economy because 84% of it is consumed locally and 16% is used as a raw material in the industries [9,20]. Kormawa and Akoroda [24] and Nwokoro et al. [30] noted that where other food crops fail, the drought-tolerant cassava crop thrives on marginal soils with a moderate climate. However, Nigeria's recent extreme weather events, including flooding, a protracted drought, higher temperatures, and variable rainfall, have exposed cassava production to the negative effects of climate change.

There is an evidence that climate change has caused an increase in pest and disease activity, slowed growth, discoloration of cassava roots and leaves, and reduced tuber production [3]. This unpleasant incidence has also increased the rate of tuber decays and pre-harvest losses in cassava farming [26] and inadvertently raised the unit cost of cassava derivatives [12,36]. Consequently, farmers pay dearly through low productivity and profitability when these disasters hit [,[16], [36]]. Thus, rural farmers need to be prepared to embrace adaptation measures that can help to realize a climate smart agriculture in order to cope with the effects of the persistent climate change.

Through climate smart agriculture, the Nigerian government has been working with the Food and Agriculture Organization (FAO) and International Crop Research Institute for the Semi-Ari Tropics (ICRISAT) to increase smallholders' ability to respond to climate change [42]. Thus, without considering the relative contributions to crop productivity, the use of improved crop varieties, crop diversification, irrigation, multiple cropping, multiple planting dates, and soil conservation practices, among others, has gained traction in practice [29,36]. This is mainly because these practices are “adaptively” accurate, relatively accessible and compatible with the already-existing local adaptation knowledge.

Several research [17,19,33] have investigated how adaptation to climate change affects cassava output. Endowments like household size, farm size, access to credit, ownership of livestock, farm and non-farm income, and capital resources all play a significant role in influencing the choice of the most effective climate change adaptation practice in cassava farming [31,36,43]. However, only a few studies have critically examined the impact of farmers' adaptation strategies to climate change on cassava productivity[16]. Hence, this study aims to (i) describe socioeconomic characteristics of cassava farmers (ii) profile choice of adaptation practices used to combat climate change in cassava farming; (iii) identify factors influencing the choice of climate change adaptation practices; and (iv) explore the effects of these practices on cassava productivity in Osun State, Nigeria.

## 2 Materials and methods

**Fig. 1:**
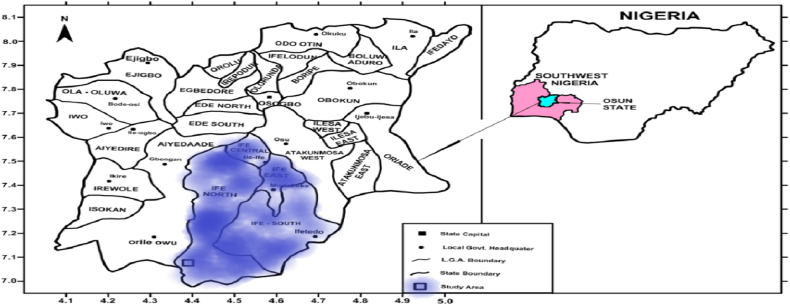
Map of the study area. Adapted from Faleyimu et al. [44].

The study was carried out in Osun State, southwestern Nigeria (Fig. 1). The State is located between latitudes 7.0° North and 9.0° North of the equator and longitudes 2.8° East and 6.8° East of the meridian. Osun State lies in the equatorial rainforest belt. It has a land area of about 8521 km^2^ and a projected population of approximately 4,435,800 people [28]. The State lies between 300 m and 600 m above sea level with a largely gentle and undulating landscape. Osun State has a tropical climate with weather conditions varying in-between the rainy season (March–October) and the dry season (November–February). The wet season is characterized by the annual rainfall ranges in-between 1500 mm and 3000 mm while the dry season is characterized by the cold dry winds and harmattan dust from the northern hemisphere. Also, the rainy season is associated with Southwest monsoon wind from Atlantic Ocean while the dry season witnesses deserts blow into the southern region associated with the Northeast trade wind from Sahara desert. Osun State has a fresh mangrove forest with the lowland stretching towards the Northern boundary [44]. Agriculture, which is the primary occupation, accounts for over 70% of the State's labour force [27]. The good soil and favourable climate is suitable for the cultivation of crops such as cassava, yam, maize, oil palm, plantain, and livestock husbandry.

The study was a field survey of cassava farmers between March and April 2021. An open data kit (ODK) questionnaire was administered by a trained interviewer by reading out loud the questions from the questionnaire on a mobile device. The information gathered included the socioeconomic attributes of cassava farmers, cassava output, size of farmland cultivated to cassava, climate variability indicators, and the common climate change adaptation practices in cassava farming. The study employed a multi-stage sampling technique to obtain relevant cross-sectional data on cassava farming and climate change in Osun State. The first step involved the purposive selection of the rain forest out of the three agricultural ecological zones (AEZ) in the state. At the second stage, four (4) Local Government Areas in Ife Community were purposively selected due to the effective production of cassava in the zone (Osun State Ministry of Agriculture and food Security). These were Ife Central, Ife North, Ife South and Ife East Local Government Areas. At the third stage, 30 smallholder cassava farmers were selected from each of the four (4) Local Government Areas using the Snowball sampling technique to give a total of 120 farmers. However, only 100 of the returned questionnaires contained relevant information for analysis.

Descriptive statistics, multivariate probit, and the generalized linear regression models were used to examine the study objectives. Descriptive statistics identified the socioeconomic traits of the farmers and profiled the adaptation practices chosen by the farmers to resist climate change. The multivariate probit regression model addressed factors influencing the choice of climate change adaptation techniques among cassava, whereas, the generalized linear regression model captured effects of climate change adaptation methods on cassava productivity. The variance inflection factor and contingency coefficient for continuous and dummy explanatory factors were used to test for multicolinearity among the predictor variables, but in both tests, multicolinearity was not shown to be an issue.

### 2.1 Ethical clearance for the study

Ethical approval for this study was sought from the cassava farmers’ association and community leaders in Ile-Ife for permission to sample their members in the study. Furthermore, expression of consent was obtained from the participant farmers after completing a consent form that explained the purpose of the study and the potential benefits. The farmers were also made to understand that participation is completely voluntary, information obtained would be treated with the utmost confidentiality and they have the right to withdraw from participation if so desired.

### 2.2 Theoretical foundation and conceptual framework

#### 2.2.1 Rational choice theory of utility maximization and random utility theory

The rational choice theory of utility maximization uses an individual method to simulate how an economic actor might behave while making a choice. In his book “Wealth of Nation”. Adams Smith advanced the idea that people have a propensity for making self-interested decisions that could engender prosperity. In other words, people act in their own best interests by making decisions that are consistent with their highest-benefit goals. This suggests that a rational economic agent will choose option(s) whose advantages outweigh the costs among the available alternatives in order to maximize economic rewards with the constrained resources at his/her disposal. This theory holds that the decisions made by economic agents are their own. This means that if the relative benefits decrease below the incurred costs, either no choice decision will be made or an alternate option or options of comparable benefits will be selected. Therefore, the decision-making process is not influenced by outside forces like coercion, environmental pressure, cultural tradition, or unconscious motivation. This is because the rational choice theory does not take into consideration instinctive behavior, intuitive reasoning, or fixed learning principles, which may potentially influence decision-making under larger costs [2], meaning this theory has a limited range of applications. Notwithstanding, rational utility theory is appropriate for evaluating how cassava farmers behave in order to maximize productivity in connection with their selection of climate change adaptation practices while operating within a constrained budget.

#### 2.2.2 Modeling discrete choice analysis (DCM)

**Fig. 2:**
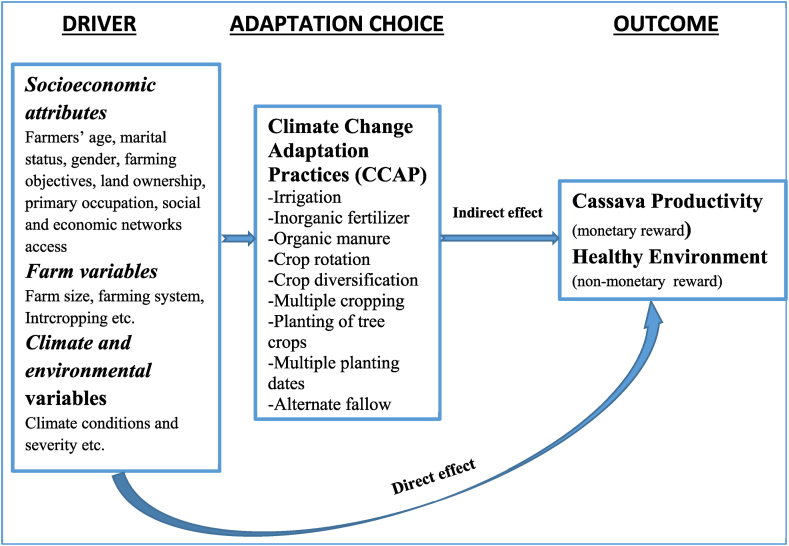
Conceptual framework for the choice of climate change adaptation practices in cassava farming and its effect on productivity.

Depending on individual farmer's farming goals, the main goal of climate adaptation practices may be economic and/or non-economic (Fig. 2). Given the values of the qualities and alternatives with higher utility, an economic agent will choose climate change adaptation methods in accordance with the random utility theory [2]. A rational cassava farmer is expected to select a set of adaptation strategies that have lower costs but greater productivity potentials in order to maximize the economic reward of adaptation in cassava farming, even if these activities have adverse effects on the environment. When making adaptation decision, it will be irrational, and hardly possible, for a smallholder farmer to prioritize soil preservation and fertility enhancement options over profit maximization objective. However, increased farming rewards are possible if a farmer prioritizes improving both the crop's productivity and the benefits to the environment when choosing adaptation practice(s). This increases the likelihood of selecting multiple adaptation practices to maximize farming profits.

In this study, relative productivity serves as the basis for evaluating cassava farmers' decisions to use specific climate change adaptation strategies. It is believed that farmers do not choose the adaptation strategies based on socio-economic attributes only, but also the farm capacity, farming system(s), and climate variables (Fig. 2). These drivers of adaptation choices can have a direct influence on farm productivity (economic goal of adaption) and environmental health (a non-economic goal of adaptation). It is anticipated that the farmers' choice of climate change adaptation practice(s) will also indirectly improve or worsen both the farms' environmental conditions and cassava productivity. This study therefore focuses on determining the factors influencing choice of climate change adaptation practices and identifying the best climate adaption techniques that could sustainably increase Nigeria's cassava productivity and support climate smart agriculture.

### 2.3 Econometric analysis

#### 2.3.1 Specification of multivariate probit (MVP) model

Discrete choice or discrete multivariate models can be used to model preference and adaptation practice under random utility theory. A random utility function allows for a partial analysis of the chosen practice over all other exclusive alternatives. When dealing with multivariate normal data, Maddala [35] and Greene [32] suggest the use of the Multivariate Probit (MVP) model to explain the determinants of random choice practices of exclusive alternative binary options. MVP model is a link between a deterministic model and a statistical model of human behavior. In this study, farmers' decisions to choose or not to choose specific adaptation practices are specified as a linear function of the farmer's specific characteristics and farm attributes (X), as shown below.

Assuming that there are *i* = (1, …,N) farmers deciding whether or not to practice $K_{j}$ = ($K_{i},...,K_{n}$) adaptation options on their cassava farm (f), where, k could be taken as jth choice of adaptation practice and *i* as the individual farmer, We can let $Y_{ikj}$ symbolizes the benefits of adopting the climate change adaptation practices (Kj): Kj = representing choice protfolio such as crop diversification (Cd), irrigation (Ir), inorganic fertilizer (If), organic fertilizer (Of), crop rotation (Cr), planting of tree crops (Ptc), multiple planting dates (Mpd), multiple cropping (Mc), and alternate fallowing (Af). Cassava farmer will decides to adopt the jth adaptation practice if $Y_{i\mathrm{fkj}}^{*}$ = $Z_{\mathrm{kj}}$ >0. It is assumed that the net benefit $Y_{i\mathrm{fkj}}^{*}$ obtained by the cassava farmer from the selected adaptation practice on their farm plot (f) is assumed to be a linear combination of observed farm and farmer-specific characteristics ($X_{if}$) and unobserved farm and farmer specific characteristics ($e_{if}$) known as the stochastic error term.

(1) $$Y_{if\mathrm{kj}}^{*}=\beta_{i}x_{if}+\varepsilon_{if}\;where\;\left(k=Cd,Ir,If,Of,Cr,Ptc,Mpd,Mc,Af\right)$$

where β is the parameter of the observed characteristic (X) to estimate. The unobserved preferences in Eq. (1) convert into the observed binary outcome equation for each option as follows, given the indicator function:

(2) $$Y_{i\mathrm{kj}}=\left\{ \begin{array}{c} 1\;if\;Y_{if\mathrm{kj}}>0\\ 0\;otherwise \end{array}\right.$$

Considering the latent nature of the estimation (Eq. (2)), which depends on observable binary discrete variables ($Y_{if\mathrm{kj}}$) that represent whether a farmer (i) chooses to practice a certain climate adaptation method (kj) on the farm (f) or not, Eqs. (1), (2) specify univariate probit models. In this model, information on farmers' adopting one farming practice does not affect the prediction of the probability that they will adopt another practice. This is the case if adoption of a particular practice is independent of whether or not a farmer adopts another practice (i.e., if the error terms, $\varepsilon_{if}$ are independently and identically distributed with a standard normal distribution). A more realistic specification, however, is to suppose that the error terms in Eq. (1) collectively follow a multivariate normal (MVN) distribution, with a zero conditional mean and variance normalized to unity, where $\varepsilon_{if}$ ∼ MVN ((0, e)) and the covariance matrix U. Therefore, the error terms jointly follow a multivariate normal distribution (MVN) with zero conditional mean and variance normalized to unity in this multivariate model, where the application of various climate change adaptation solutions is available (for identification of the parameters) where, $\varepsilon_{Cd},\varepsilon_{If}$ $\varepsilon_{Ir}$, $\varepsilon_{If}$, $\varepsilon_{Of}$, $\varepsilon_{Cr}$, $\varepsilon_{Ptc}$, $\varepsilon_{Mpd}$, $\varepsilon_{Mc}$, $\varepsilon_{Af}$ and the symmetric covariance matrix ∑ is given in Eq. (3) below.

(3) $$\sum =\left[\begin{array}{ccccc} 1 & \rho_{12} & \rho_{13} & \cdots & \rho_{1c}\\ \rho_{21} & 1 & \rho_{22} & \cdots & \rho_{2c}\\ \rho_{31} & \rho_{32} & 1 & \cdots & \rho_{3c}\\ \vdots & \vdots & \vdots & 1 & \vdots \\ \rho_{c1} & \rho_{c2} & \rho_{c2} & \rho_{c2} & 1 \end{array}\right]$$

The covariance matrix's off-diagonal parts, which stand for the unobserved correlations between the stochastic elements of the selection of adaptation strategies, are of particular relevance. According to this supposition, Eq. (2) produces the MVP model, which collectively embodies decisions on whether to opt for a particular adaptation practice or not. This specification with non-zero off-diagonal elements permits correlation between the error terms of various latent equations, which represent unobserved features that influence the selection of alternative adaptation options (Table 1). The null hypothesis that the correlation coefficients (ρ statistics) are jointly equal to zero against the alternative that rho (ρ) are not jointly equal to zero were tested using a likelihood ratio method (Table 1).

**Table 1:** Correlation coefficient likelihood ratios.

	$\rho_{Cd}$	$\rho_{Ir}$	$\rho_{If}$	$\rho_{Of}$	$\rho_{Cr}$	$\rho_{Ptc}$	$\rho_{Mpd}$	$\rho_{Mc}$	$\rho_{Af}$
$\varepsilon_{Cd}$	1.0000								
$\rho_{Ir}$	0.3480	1.0000							
$\rho_{If}$	0.3042	0.2629	1.0000						
$\rho_{Of}$	0.4033	0.0491	0.5579	1.0000					
$\rho_{Cr}$	0.3313	0.0755	0.2912	0.2484	1.0000				
$\rho_{Ptc}$	0.1612	0.4491	0.2949	0.0693	0.1553	1.0000			
$\rho_{Mpd}$	0.0795	0.1740	0.3257	0.1485	0.1035	0.0389	1.0000		
$\rho_{Mc}$	0.1843	0.2801	0.3269	0.2653	0.2000	0.3096	0.1801	1.0000	
$\rho_{Af}$	0.2712	0.2666	0.2412	0.2513	0.0225	0.3584	0.2237	0.2441	1.0000

Likelihood ratio test of rho21 = rho31 = rho41 = rho51 = rho32 = rho42 = rho52 = rho43 = rho53 = rho54 = 0: chi2(36) = 250.410, Pr > chi2 = 0.0000.

Source: Authors' computation, 2021.

The results significantly invalidate the idea that the error terms are independent of one another, supporting the use of MVP.

#### 2.3.2 Generalized linear regression model (GLRM)

Cassava farmers' decision to adopt a practice was anticipated to depend on the benefits that may be derived from the practices under the rational choice theory of utility maximization (U). To determine the productivity of cassava farming under different climate change adaptation practices, the GLRM model was used. This model was employed due to its reliability when taking into account response variables having error distributions different to normal distribution [5]. A distribution model for data observations can be chosen using GLM based on the understanding of the data. This is made possible by the link function, which reduces heteroskedasticity in datasets by converting the mean of data observations, E[Yi], into a linear form [14]. In other words, the conical form distribution options allow for the selection of data randomness, linearity, and link functions, which means that the variance of errors in Yi needs not be constant when using GLRM [5,14]. The GLRM problem has traditionally been approached using a linear regression model.

(4) $$\gamma_{j}=\alpha +\beta x_{j}+e_{j}$$

where the γ_j_ is the cassava productivity, x_j_ are farmers' socio-economic and climate attributes, j = l, …, n observed, α + β unknown parameters of interest, the ε_j_ is a zero mean error variate, with the x_j_ p column-vectors, and a β p row-vector.

If the mean of x in Eq. (5) is

(5) x‾=∑j=1nxjn

the ordinary least squares estimate in Eq. (6), S, satisfies

(6) $$\hat{B}\sum_{j}\left(x_{j}-\bar{x}\right){\left(x_{j}-x\right)}^{\tau }=\sum_{j}y_{j}{\left(x_{j}-\bar{x}\right)}^{\tau }$$

where τ denotes the operation of matrix transposition. It is demonstrated that, in the case where the regressors are joint “Gaussian,” the ordinary least squares estimates have a working interpretation for a broader class of models than one might have imagined. The solution, β and γ, of Eq. (4) is shown to provide an estimate of α in the model up to an unknown constant of proportionality. Equation (7) explicitly describes GLRM as follows.

(7) $$U_{i}=\alpha_{i}+\sum_{i=1}^{n}\left({\beta_{ij}X}_{ij}+e_{i}\right)$$

where: Ui = utility derivable (productivity) of ith farmer due to the practice of climate change adaptation practices; Xj = choice(s) of adaptation practices which are diversification, irrigation, inorganic fertilizer, organic manure, crop rotation, planting of tree crops, multiple planting dates, multiple cropping, alternate fallowing and farmers' socio-economic control variables which are age (age square), household size, education level, membership of cooperative societies, credit access, farming experience and access to extension visit of ith cassava farmer; β_i_ = Coefficient to be estimated and e_i_ = the error term. The assumption is that the error term e_i_ is normally distributed with a mean of zero and the variance is one, e_i_ – N (0,1).

The adaptation practices are dummy variables taking a value of 1 or 0. Table 2 shows the description of the dependents and the independent variables for the analyses.

**Table 2:** Description of dependent and independent variables.

Variable	Options	Unit of measurement (data type)
Adaptation practices	Irrigation, inorganic fertilizer, organic manure, crop rotation, crop diversification, multiple cropping, planting of tree crops, multiple planting dates, alternate fallow	1 = yes, 0 = no, for each practice (Dummy)
Cassava productivity	Cassava output per unit area	Kilogramme per hectare (continuous, ratio)
Age	Age of cassava farmers	Year (count)
Gender	Sex of cassava farmers	1 = Male, 0 = female (dummy)
Marital status	Marital status of the cassava farmer	1 = married, 0 = otherwise (dummy)
Household size	Number of household members per farmer	Number (count)
Level of education	Years spent to acquiring formal education	Years (continuous)
Primary occupation	The primary occupation of cassava farmers	1 = farming, 0 = otherwise (dummy)
Income	Total income earned from cassava farming	Naira (continuous)
Cooperative society	Membership in cooperative society	1 = belong, 0 = not belong (dunny)
Years of experience	Number of years farmers had been involved in cassava farming	Year (continuous)
Farm size	Area of land cultivated to cassava	Hectare (Continuous)
Farming objectives	Objective of cassava farming	1 = profit, 2 = consumption, 3 = both (discrete)
Start-up capital	Amount invested in starting up cassava farm	Naira (continuous)
Access to credit	Access to credit by cassava farmers	1 = access, 0 = no access (dummy)
land ownership	Method of land acquisition	1 = purchased, 2 = inherited, 3 = leased, 4 = gifted (discrete)
Extension visit	Access to extension agent visit	1 = yes, 0 = no (dummy)
Climate info	Frequency of climate information	4 = very frequent, 3 = frequent, 2 = occasionally, 1 = rarely (categorical)
Farming system	System of farming used in cassava farms	1 = mono-cropping, 2 = mixed cropping (categorical)
Inter-cropping	inter-cropping cassava and other crops	1 = yes, 0 = no (dummy)
Climate severity	Severity of climatic condition	1 = increased, 0 = reduced (dummy)
Climate condition	Knowledge of climatic condition	1 = good, 2 = fair, 3 = moderate (categorical)

Author's computation, 2021.

## 3 Results and discussion

### 3.1 Socio-economic characteristics of the farmers

Result in Table 3 shows that the mean age of the farmers was 45.46 ± 9.36 years, this implies that the farmers were in their active and economic age, that have adaptive and innovative capacities for climate change adaptation practices [38]. Male gender accounted for 78% of the farmers (Mean score = 0.78 ± 0.42), showing that cassava production is male dominant. This agrees with [36] who noted male gender are more involved in cassava production in Southwestern Nigeria than the female gender. However, it negates [15] who stated that cassava production is female dominated due to high urban migration of the male gender. About 77% of the farmers were married (Mean score = 2.11 ± 0.58) with an average household size of 5.07 ± 2.02 members. Table 3 also reveals that cassava farmers were fairly educated as the majority of the farmers had at least primary school education, spending an average of 6.09 ± 1.39 years in acquiring formal education. This indicates that cassava farmers are considerably literate and should be enlightened to adopt new climate adaptation strategies. This submission supports the findings of [36]. The mean years of experience in cassava farming was 13.71 ± 10.29 years. The result agrees with [36] that cassava farmers have 10 years average experience in farming. Obayelu et al. [31] further explained that judicious use of resources and efficient management of farm activities are usually influenced by the accumulated years of experience of the farmer. Majority (66%) of the farmers belonged to a cooperative society (Mean score = 0.66 ± 0.49). Invariably, cassava farmers are more likely to enjoy inclusive benefits membership of these trade networks provide which include access to the financing incentives such as loans, discounted prices of inputs, and farming related information. This submission is in line with the findings of [8] that farmers leveraging on the benefits of cooperative society tend to adopt various climate adaptation strategies.

**Table 3:** Socio-economic characteristics of cassava farmers.

Variables	Mean score	Standard deviation
Age	45.46	9.36
Male gender proportion	0.78	0.42
Marital status	2.11	0.58
household size	5.07	2.02
Level of education	6.09	1.39
primary occupation	2.50	1.05
Income	149,900	76,719
cooperative society	0.66	0.49
Years of experience	13.71	10.29
Farm size	2.02	1.09
Farming objectives	0.85	0.36
Start-up capital	1.48	0.85
Access to credit	0.67	0.48
land ownership	2.59	1.102
Extension visit	0.62	0.50

Source: Author's field survey, 2021.

About 70% of the farmers used personal savings to start cassava farming (Mean score = 1.48 ± 0.85), while about 67% had access to credit (Mean score = 0.67 ± 0.48). The average cassava farm size was 2.02 ± 1.09 ha. Most of the cassava farmers inherited their farmlands, therefore, farmers had some levels of rights on the use of farmland. This corroborates the findings of [7]. Table 3 further indicates that an average of 0.62 ± 0.50, that is 62% of the farmers, had access to extension services. This implies that the majority of the farmers have a better likelihood of getting information that could improve cassava productivity premised on the access to extension services. Agriculture was the primary occupation (Mean score = 2.50 ± 1.05) of the farmers with an average income of ₦149,000±₦76,000. Majority (85%) of the Cassava farmers produced crops mainly for profit (Mean score = 0.85 ± 0.36), suggesting the importance of identifying best adaptation strategies that could boast cassava productivity.

### 3.2 Choice of adaptation practices used to combat climate change in cassava farming

Table 4 shows that the major adaptation practices among the cassava farmers were organic fertilizer application, crop diversification, multiple crop varieties and inorganic fertilizer.

**Table 4:** Choice of adaptation practices to combat climate change in cassava farming.

Adaptation practice	Not practised	Practised			WMS	SD	T-STAT	Cumulative Remark
		NE (1)	ME (2)	VE (3)				
Crop diversification	41 (41%)	0 (0%)	34 (34%)	25 (25%)	2.42	.498	37.354	E
Irrigation practices	77 (77%)	0 (0%)	15 (15%)	8 (8%)	2.35	.486	23.121	E
Inorganic Fertilizer application	36 (36%)	0 (0%)	42 (42%)	22 (22%)	2.34	.478	39.167	E
organic manure application	23 (23%)	0 (0%)	52 (52%)	25 (25%)	2.32	.471	43.280	E
Crop rotation	57 (57%)	0 (0%)	18 (18%)	25 (25%)	2.58	.499	33.911	E
Planting of tree crops	84 (84%)	7 (7%)	4 (4%)	5 (5%)	1.88	.885	8.474	NE
Multiple planting dates	84 (84%)	0 (0%)	12 (12%)	4 (4%)	2.25	.447	20.125	E
Multiple crop types/improved crop varieties	76 (76%)	0 (0%)	17 (17%)	7 (7%)	2.29	.464	24.180	E
alternate fallowing	77 (77%)	1 (1%)	16 (16%)	6 (6%)	2.21	.518	20.512	E
Land fragmentation	99 (99%)	0 (0%)	0 (0%)	1 (1%)	3.00	–	–	–

**Note:** E: effective, VE: very effective, ME: moderately effective, NE: not effective**.**

Cumulative remark**:** E ≥ 2.0 and NE < 2.0.

Source: Author's field survey, 2021.

Organic manure application was the most commonly adopted practice among the cassava farmers. The practice was adopted by 77% of the farmers with a weighted mean score (WMS) of 2.32 ± 0.471 (t = 43.280, *p* < 0.01), asserting that organic manuring was perceived to be an effective practice among the cassava farmers. Similarly, 64% of the farmers adapted inorganic fertilizer application. The effectiveness of this practice had a WMS of 2.34 ± 0.478 (t = 39.169, *p* < 0.01). Cumulatively, about 59% of the farmers practiced crop diversification as an adaptation strategy (WMS = 2.42 ± 0.498; t = 37.354, *p* < 0.01). However, few of the farmers practiced irrigation (23%), alternate fallowing (23%), planting of trees (16%), multiple planting dates (16%) and multiple crop types/improved varieties (24%). This findings are similar to the study by Owoeye [36] that analyzed farmers’ choice of climate change adaptation methods in the southwestern Nigeria.

### 3.3 Socio-economic and climatic factors influencing the choice of climate change adaptation practices among cassava farmers

In Table 5, the parameters of multivariate probit regression analysis gave coefficients of determination (R^2^) ranging between 0.215 and 0.4326 with F-statistics of 1.562–3.677. This indicates that the models had good fits with about 21%–43% of the variations in the choice of climate change adaptation practices, explained by the predictor variables in the models.

**Table 5:** Multivariate probit (MVP) regression analysis of socio-economic and climatic factors influencing the choice of climate change adaptation practices among cassava farmers.

Variables	Diversification	Irrigation	Inorganic fertilizer	Organic manure	Crop Rotation	Planting of tree crop	Multiple planting dates	Multiple crop types/Improved crop varieties	alternate fallowing
Age	0.118	0.011	−0.267	−0.061	−0.264	−0.065	−0.555***	−0.145	−0.125
Gender	−0.066	−0.132	0.202	0.014	−0.051	−0.167	0.226*	−0.391**	0.050
Years of education	0.008	0.023	−0.011	−0.067**	−0.044	0.006	−0.000	0.055	0.027
primary occupation	−0.069	−0.101	0.142***	0.077	0.037	−0.001	0.077*	−0.019	−0.015
Income	0.098	−0.014	0.056	0.006	−0.105	−0.278***	0.072	−0.013	−0.072
Coop Membership	0.050	0.055*	0.188	−0.133	−0.038	−0.115	−0.022	0.371**	−0.193
Farming experience	−0.092	−0.051	0.242***	−0.008	0.256**	−0.028	0.100	0.104	0.065
Farming objective	0.530***	0.051	0.252	0.344**	0.005	0.182	−0.063	0.006	0.159
Access to credit	0.014	−0.005	0.018	0.038***	0.034***	0.005	0.008	−0.006	0.025**
Mode of land ownership	0.011	0.019	0.061	0.039	0.079*	−0.012	0.003	0.036	0.011
Extension access	−0.142	−0.087	−0.271***	−0.035	−0.033	0.064	−0.137*	−0.125	−0.056
Infomation frequency	0.047	−0.036	0.002	−0.022	0.003	0.002	−0.008	0.025	0.029
Farm size	−0.008	0.025	0.010	−0.004	−0.001	0.039***	0.005	0.020	0.017
Farming system	0.106	−0.236	−0.318	0.416*	−0.274	−0.376*	−0.165	−0.190	0.042
climatic condition	0.006	−0.002	0.017	0.011	0.030	0.017	0.035**	0.011	0.040**
severity of climate	0.100	−0.330***	−0.107	0.111	0.161	−0.088	0.037	0.104	0.136
Inter-cropping	−0.283	0.040	−0.108	−0.085	−0.424**	−0.118	0.092	−0.121	0.073
cons	−1.985	1.714	0.133	−0.736	2.252	4.296***	0.946	0.556	0.098
R-square	0.2446	0.2744	0.3465	0.4326	0.3372	0.4026	0.3266	0.2503	0.2150
F-statistics (p-value)	1.561872*	1.824**	2.557*	3.677***	2.454***	3.251***	2.339***	1.610*	1.562

Author's computation, 2021. Values in parenthesis are the standard error. ***p < 0.01, **p < 0.05, *p < 0.1.

Note: data used 500 replications to bootstrap the standard errors after changing bootstrap replications between 100 and 1000 with no significant changes.

Table 5 shows that the marginal coefficients of crop diversification (β = 0.053, *p* < 0.01) was statistically significant and positively correlated with the farming objectives of the farmers. Thus, 1% change in farming objective of the farmers enhances the tendencies that crop diversification will be practiced among the cassava farmers by 53%. This means that farmers whose farming objective are tilted more readily towards consumption would be more likely to practice crop diversification to reduce crop loss due to adverse climatic condition than those that aim at making profit from farming.

Irrigation practice was significantly and positively influenced by membership of cooperative society (β = 0.055, *p* < 0.1) but negatively influenced by climate severity (β = −0.330, *p* < 0.01). Table 5 reveals that if there is 1% increase in membership of cooperatives, the possibility that cassava farmers will practice irrigation will grow by 5.5%. In contrary, 1% increase in climate severity will result to 33% decrease in irrigation practice. This implies that cooperative groups encourage cassava farmers to use irrigation, either through loan financing or collective actions; and irrigation is an appealing adaptation option in cassava cultivation when climatic circumstances are favourable. This contribution concurs with [34], who observed that although farmers are fully aware of climate change, they are unable to implement adaptation practices due to a lack of financial resources and access to weather data.

Inorganic fertilizer was statistically significant and positively influenced by the marginal coefficients of the use of primary occupation (β = 0.142, *p* < 0.01) and farming experience (β = 0.242, *p* < 0.01), but negatively influenced by access to extension visit (β = −0.271, *p* < 0.01). Result in Table 5 shows that a unit increase in the primary occupation (from non-farming to farming) and number of years that a farmer has been involved in cassava farming will result to 14.2% and 24.2% increase while 1% increase in extension access will yield 27.1% decrease in the livelihood of practicing inorganic fertilizer. This suggests that experienced cassava farmers and those taking farming as a major occupation, are more likely to choose inorganic fertilizer to reduce the effects of climate change than their less-experienced counterparts, who are primarily into non-farming occupations. The inverse relationship between inorganic fertilizer and access to extension visit suggests that farmers who interact with extension agents are swayed from the use of inorganic fertilizer as a method of coping with climate change, most likely as a result of the disruptive effects of excessive use of inorganic fertilizer on the environment. This finding is consistent with [38], who noted that recent development in extension service aims at discouraging farmers from using inorganic fertilizer because of the associated environmental implications. This implies that farmers who have access to extension services are likely well-informed about the detrimental effects of applying inorganic fertilizer when growing cassava on the environment. As a result, extension access might be a useful strategy for encouraging cassava farmers to adopt climate smart agriculture.

For the organic fertilizer, marginal coefficients of credit access (β = 0.038, *p* < 0.01) and farming objectives (β = 0.344, *p* < 0.05) were statistically significant and positively correlated while years of education (β = −0.067, *p* < 0.05) was negatively correlated. Result shows that 1% increase in access to credit and farming objectives will improve the probability that organic fertilizer will be practiced by 3.85% and 34.4%, respectively. On the other hand, 1% increase in the years of education will reduce the livelihood of practicing the use of organic fertilizer by 6.7%, implying that educated farmers are less likely to use organic fertilizer than the non-educated counterparts. According to Salue et al. [37], there is a poor awareness and inadequate technical understanding of organic fertilizer application among smallholder farmers in Nigeria. This could serve as disincentive to the practice of organic fertilizer in cassava farming.

The determinants of the crop rotation practice are farming experience (β = 0.256, *p* < 0.05), credit access (β = 0.034, *p* < 0.01), land ownership (β = 0.079, *p* < 0.1) and inter-cropping (β = −0.424, *p* < 0.001). If there is 1% increase in the years of experience in cassava farming, land ownership, and access to credit, the probabilities that cassava farmers will practice crop rotation as a climate change adaptation option increases by 25.6%, 3.4% and 7.9%, respectively. On the other hand, 1% increase in the practice of intercropping will reduce the practice of crop rotation as a climate adaptation option considerably by 42.4%. This suggests that farmers that have many years of experience, access to credit, own farm land by inheritance and do not intercrop are more incentivized to practice crop rotation than the new comers, those that intercrop, and those that could not access credit and land by inheritance.

Planting of tree crops was statistically significant and positively determined by marginal coefficient of farm size (β = 0.039, *p* < 0.01), but negatively correlated to farm income (β = −0.278, *p* < 0.01) and farming system (β = −0.376, *p* < 0.1). This implies that a unit increase in farmers’ income and the farming system (mono-cropping to a mixed-cropping) will decrease the likelihood of planting tree crops by 27.8% and 37.6%. On the other hand, a unit increase in the size of farms will increase the adoption of tree crop planting as a climate adaptation practice by 3.9%. It thus appears that farmers that cultivate large farms, earn low income, and use mono-cropping system are more inclined to planting tree crops than those that cultivate small farms, earn huge income and use mixed cropping system. This suggests that tree crops are typically grown on large farms when there is insufficient funding to employ alternative adaptation practices in addressing climate challenges. It is therefore inferred that planting of tree crops is not thought of as an effective climate change adaptation option in cassava farming because when large cassava farmers experience an increase in income, there is a high tendency that tree crop planting will be abandoned in favour of other climate change adaptation options. This supports the finding in Table 4 that growing tree crops is a rare practice and ineffective in the cassava farming industry. This submission backs up the findings of [11], who noted that farm size influences adaption choices in a positive way.

Multiple planting dates was statistically significant and positively correlated with marginal coefficient of gender (β = 0.226, *p* < 0.1), primary occupation (β = 0.077, *p* < 0.1), and climatic condition (β = 0.035, *p* < 0.05) but negatively correlated with age (β = −0.555, *p* < 0.01) and access to extension agents (β = −0.137, *p* < 0.1). The result indicates that a positive change in primary occupation (from non-farming to farming), gender (from female to male) and climatic condition will increase the probability of adopting multiple planting dates by 7.7%, 22.6% and 3.5%, respectively. However, 1% increase in age and access to extension visits will decrease the practice of multiple planting dates by 55.5% and 13.7%, respectively. The older farmers are accustomed to the planting dates utilized in the past and are less ready to take production risks than younger farmers, which may explain the negative link between farmer age and multiple planting dates usage. It was discovered that male cassava growers are more inclined than their female counterparts to the use multiple planting dates. This supports the finding in the study of [41] that female-headed households rarely practice multiple planting dates.

Practice of multiple crop types/improved crop varieties was statistically significant and positively correlated to the marginal coefficient of membership of cooperative societies (β = 0.371, *p* < 0.05) but negatively correlated to gender (β = −0.391, *p* < 0.05). Congruently, a 1% increase in membership of cooperative society will increase the use of multiple crop types/improved crop varieties by 37.1%. On the other hand, a change in gender (from female to male) will result in a drop in multiple crop types/improved crop varieties as a climate adaptation technique, suggesting that men are less likely than women to plant multiple crop types/improved varieties. There is a likelihood that cooperative organizations frequently assist farmers in obtaining improved cassava varieties from research institutions. Therefore, joining cooperative groups might be essential to enhancing male cassava producers' use of multiple crop types/improved varieties as an adaptation option.

Alternate fallowing was influenced by credit access (β = 0.025, *p* < 0.05) and climatic conditions (β = 0.040, *p* < 0.05). The result revealed that 1% increase in credit access and climatic conditions would increase the likelihood of practicing alternate fallowing by 2.5% and 4%, respectively. This demonstrates how the availability of capital and a favourable climate condition influence the practice of alternate fallowing. Therefore, resource-poor cassava farmers might consider alternate fallowing as an unappealing adaptation option because it can only be used under better climatic conditions.

### 3.4 The effect of climate change adaptation practices on cassava productivity

The generalized linear model (GLM) with a Gaussian regressor model parameters showed that Akaike criteria (AIC) was 302.238 and significant (*p* < 0.01), indicating that the model had a good fit and suitable for the analysis (Table 6). Table 6 shows that cassava productivity was significantly and positively influenced by the practice of alternate fallowing (β = 0.461, *p* < 0.1), organic fertilizer practice (β = 0.919, *p* < 0.1) and use of multiple crop types/improved crop varieties (β = 0.126, *p* < 0.05) but negatively influenced by multiple planting dates (β = 0.648, *p* < 0.1), the system of farming (β = 0.242, *p* < 0.01) and crop diversification (β = − 0.496, *p* < 0.01). Furthermore, years of education (β = 0.185, *p* < 0.5) and access to credit (β = −0.847, *p* < 0.5) influenced productivity.

**Table 6:** Effect of climate change adaptation practices on cassava productivity.

Productivity	Coef.	St.Err.	p-value	Sig
Alternate fallowing Multiple planting date Organic manure System of farming Diversification	0.461 −.648 .919 −.68 −.496	.269 .38 .471 .264 .185	.086 .088 .051 .01 .007	* * * *** ***
Multiple crop types/Improved crop varieties	.126	.049	.01	**
Irrigation	.178	.181	.326	
Inorganic Fertilizer application	.487	.305	.111	
Crop rotation	.282	.268	.294	
Tree cropping	−.256	.269	.34	
Multiple cropping	−.079	.171	.645	
Power (Strength)	−.232	.285	.415	
Gender	−.21	.239	.379	
Household size	.2	.135	.137	
Years of Education	.185	.083	.026	**
Cooperative membership	−.114	.252	.65	
Credit access	−.847	.351	.016	**
Farming experience	−.008	.012	.534	
Access to extension visit	−.241	.168	.151	
Constant	10.03	.599	0	***
Mean dependent var	9.855			
Number of obs	100	SD dependent var:		1.100
Prob > chi2	0.003	Chi-square:		40.276

***p < 0.01, **p < 0.05, *p < 0.1.

Author's computation, 2021.

Note: data used 500 replications to bootstrap the standard errors after changing bootstrap replications between 100 and 1000 with no significant changes.

Result showed that the coefficient of the practice of alternate fallowing, the use of organic manure, and multiple crop types/improved crop varieties influenced cassava productivity at a significant level of 10% and 5%, respectively. A unit increase in alternate fallowing would increase cassava productivity by 46.1%. According to Lenka and Lenka [25], the practice of alternate fallowing enhances the physical qualities of the soil by stratifying organic matter and also acts as an insulator by lowering temperature swings, therefore fostering the favourable environment required for optimum crop growth. The value of organic fertilizer as one of the finest adaptation methods is further demonstrated by the fact that its application improved productivity by 91.9%. This confirms the findings of [10] that the appropriate use of organic fertilizers results in high cassava yields. A unit increase in the planting of multiple crop types/improved crop varieties also raised cassava productivity by 12.6%. According to Afolami et al. [1], improved cassava varieties are stress-resistant,drought-tolerant and high yielding.

On the other hand, a unit increase in multiple planting dates reduced productivity by 64.8%, suggesting that crop yield in the research area falls when planted at different planting date intervals. This submission deviates from the *a priori* expectation. Recall that Table 5 showed a negative relationship between multiple planting dates and extension services. This suggests that producers of cassava do not use correct timing, perhaps as a result of limited access to climate information when planting decisions are made [13]. In a similar manner, crop diversification and farming systems had a negative impact on cassava productivity at a significant threshold of 1%, meaning that increasing these factors by 1% will reduce cassava productivity by 68% and 49.6%, respectively. This suggests that the practice of crop diversification as a method of coping with climate change has a negative impact on cassava productivity. This submission supports the claim made by Olawode et al. [4] that crop diversification decreases crop productivity.

In addition, coefficient of education, a measure of farmers’ socioeconomic attribute had a positive correlation with cassava productivity whereas, access to credit was negatively correlated to productivity. The result showed that a 1% increase in the number of years cassava farmers spent in acquiring formal education will result in about 18% increase in cassava productivity whereas, 1% increase in access to credit reduces cassava productivity by 84.7%. This submission contradicts the a *priori* expectation and depicts likelihood of resource diversion into non-cassava based usage.

## 4 Conclusion and recommendations

The study concluded that cassava farming is male dominant and the use of organic fertilizer, crop diversification, various crop varieties, and inorganic fertilizer were the most efficient adaptation techniques for combating the effects of climate change. There were mixed effects on the factors that determined the choice of climate change adaptation practices employed in cassava farming. It was however affirmed that the goal of farming, farmer's resource endowment, access to climate information services, social inclusion, tenure security and climatic condition played significant roles in determining the choice of climate change adaptation practices in cassava farming. It was further discovered that eco-friendly climate adaptation practices including organic manuring, planting of multiple crop types/improved crop varieties, and use of alternative fallowing enhanced cassava productivity whereas, crop diversification, multiple planting dates, and farming systems inhibited it.

It is worthy of note that this study is a cross-sectional survey of Ife community, a major area for cassava farming in Nigeria. A bootstrapping method was used to generalized the result of this study. However, the findings may not be applicable to some other climes, cultures and agro-ecological spaces. The study relied primarily on the farmers’ experience for climatic information. Therefore, future research should take into account meteorological data on climate variability when examining the productivity impact of climate adaptation techniques in Nigeria.

Thereupon, this study recommended that

1) To increase cassava productivity, it is necessary to seek the integration of climatic information dissemination in extension services provided to local farmers by trade groups, agricultural agencies, and research organizations.
2) It's crucial that farmers receive training in the use of eco-friendly climate change adaptation techniques such as crop diversification, multiple planting dates, and alternate fallowing in the cultivation of cassava. Basically, cassava farmers should be urged to look into the potential of multiple crop types/improved crop varieties, organic manure application, and alternate fallowing to increase production.
3) It is important to encourage cassava farmers to join cooperative societies to optimize the use of funds accessed from credit institutions and so boost their possibilities of practicing irrigation, organic fertilizer application, and alternate fallowing.

## Author contribution statement

Olabisi Damilola Omodara: Conceived and designed the experiments; Performed the experiments; Analyzed and interpreted the data; Contributed reagents, materials, analysis tools or data; Wrote the paper.

Oluwemimo Oluwasola: Conceived and designed the experiments; Wrote the paper.

Oluwatunmise Oyebisi Afape: Conceived and designed the experiments; Performed the experiments; Contributed reagents, materials, analysis tools or data.

Oluwakemi Abosede Ige; Akinsola Temitope Oyebanji: Analyzed and interpreted the data; Contributed reagents, materials, analysis tools or data; Wrote the paper.

## Funding statement

This research did not receive any specific grant from funding agencies in the public, commercial, or not-for-profit sectors.

## Data availability statement

Data will be made available on request.

## Declaration of competing interest

The authors declare that they have no known competing financial interests or personal relationships that could have appeared to influence the work reported in this paper.

## References

[1] Afolami C.A., Obayelu A.E., Vaughan I.I. (2015). Welfare impact of adoption of improved cassava varieties by rural households in South Western Nigeria. Agric. Food Econ..

[2] Koppen M. (2001). Characterization theorem in random utility theory. Int. Encycl. Soc. Behav. Sci..

[3] Akanbi W.B., Olabode O.S., Olaniyi J.O., O Ojo A. (2004).

[4] Alawode O.O., Kabiru B.A., Akanbi A.O. (2020). Land use intensity, crop diversification and productivity of farmers in Akinyele local government area of Oyo State, Nigeria. Int. J. Innov. Environ. Stud. Res..

[5] R. Germán, Generalized Linear Model Theory. Accessed on 2 January, 2023.

[6] Antle J.M. (2008). Climate Change and Agriculture: economic Impacts. Choices-The magazine of food, farm, and resource issues. Am. Agric. Econ. Ass..

[7] Anyaegbunam H.N., Okoye B.C., Asumugha G.N. (2010). Labour productivity among smallholder cassava farmers in South East agro ecological zone, Nigeria. Afr. J. Agric. Res..

[8] Anyoha N.O., Nnadi F.N., Chikaire J. (2013). Socioeconomic factors influencing climate change adaptation among crop farmers in Umuahia South Area of Abia State, Nigeria. Neth. J. Agric. Sci..

[9] Agwu E.A., Anyaeche C.L. (2007). Adoption of improved cassava varieties in six rural communities in anambra state, Nigeria. Afr. J. Biotechnol..

[10] Ayoola O.T., Makinde E.A. (2007). Complementary organic and inorganic fertilizer application: influence on growth and yield of cassava/maize/melon intercrop with a relayed cowpea. Austr. J. Basic Appl. Sci..

[11] Belay A., Recha J.W., Woldeamanuel T., Morton J.F. (2017). Smallholder farmers' adaptation to climate change and determinants of their adaptation decisions in the Central Rift Valley of Ethiopia. Agric. Food Secur..

[12] Ceballos H., Ramirez J., Bellotti A.C., Jarvis A., Alvarez E., Yadav S.S., Redden R., Hatfield J.L., Campen H.L., Hall A. (2011). Crop Adaptation to Climate Change: Newjersey.

[13] Darand M., Masoodian A., Nazaripour H., Nazaripour M.M. (2015). Spatial and temporal trend analysis of temperature extremes based on Iranian climatic database (1962-2004). Arabian J. Geosci..

[14] Great Learning Team. Generalized Linear Models: what Does it Mean? Accessed on 7 December. 2022.

[15] Ezeibe A.B., Edafiogho D.O., Okonkwo N.A., Okide C.C. (2015). Gender differences and challenges in cassava production and processing in Abia State, Nigeria. Afr. J. Agric. Res..

[16] A Ezekiel A., Olawuyi S.O., Ganiyu M.O., Ojedokun I.K., Adeyemo S.S. (2012). Effects of climate change on cassava productivity in Ilesa-east local government area, Osun State, Nigeria. Br. J. Arts Soc. Sci..

[17] Enete A.A. (2013). Challenges of agricultural adaptation to climate change: the case of cassava post-harvest in Southeastenete Nigeria. Int. J. Clim. Change Strat. Mgt..

[18] FAOSTAT (2019).

[19] Henri-Ukoha A. (2020). Assessment of the viability of climate adaptation strategies of cassava based farmers in southern Nigeria. J. Agric. Food Sci.

[20] Ikuemonisan E.S., Mafimisebi T.E., Ajibefun I., Adenegan K. (2020). Cassava production in Nigeria: trends, instability and decomposition analysis (1970-2018). Heliyon.

[21] Stocker T.F., Qin D., Plattner G.K., Tignor M., Allen S.K., Boschung J., Nauels A., Xia Y., Bex V., Midgley P.M., IPCC (2013). Climate Change 2013: the Physical Science Basis. Contribution of Working Group I to the Fifth Assessment Report of the Intergovernmental Panel on Climate Change.

[22] IPCC (2014). Climate Change: Impacts, Adaptation, and Vulnerability. Part A: Global and Sectoral Aspects. Contribution of Working Group II to the Fifth Assessment Report of the Intergovernmental Panel on Climate Change.

[23] Ayinde O.E., Ajewole O.O., Ogunlade I., Adewumi M.O. (2010). Empirical analysis of agricultural production and climate change: a case study of Nigeria. J. Sustain. Dev. Afr..

[24] Kormawa P., Akoroda M. (2003). https://doi:10.5707/cjagricsci.2013.7.2.10.16.

[25] Lenka S., Lenka N.K. (2014). Conservative tillage for climate change mitigation-the reality. Clim. Change Environ. Sustain..

[26] Matemilola S. (2017). The challenges of food security in Nigeria. Open Access Libr. J..

[27] Medium Term Sector Strategy (2018). Agriculture sector medium term strategies. https://www.osunstate.gov.ng/wp-content/uploads/2018/11/agriculture-sector.pdf.

[28] Citypopulation. https://citypopulation.de/en/nigeria/admin/NGA030__osun/(accessed November 09, 2022).

[29] Nwaiwu I.U., Ohajianya D.O., Orebiyi J.S. (2014). Climate change trend and appropriate mitigation and adaptation strategies in Southeast Nigeria. Glob. J. Biol. Agric. Health Sci..

[30] Nwokoro S.O., Orheruata A.M., Ordiah P.M. (2002). Replacement of maize with cassava sievates in cockerel starter diets: effect on performance and carcass characteristics. Trop. Anim. Health Prod..

[31] Obayelu A.E., Olarewaju T.O., Oyelami N.L. (2014). Effect of rural infrastructure on profitability and productivity of cassava-based farms in Odogbolu local government area, Ogun State, Nigeria. J. Agric. Sci. Belgrade.

[32] Greene W.H. (1997).

[33] O.R. Ogunpaimo, A.O. Dipeolu, O.J. Ogunpaimo, S.O. Akinbode, Determinants of choice of climate change adaptation options among cassava farmers, in southwest Nigeria. FUTO J. Ser., 6(1) 25-39.

[34] Idrisa Y.L., O Ogunbameru B., Ibrahim A.A., Bawa D.B. (2012). Analysis of awareness and adaptation to climate change among farmers in the Sahel Savannah agro-ecological zone of Borno State, Nigeria. Sci. Domain Int..

[35] Maddala G.S. (1987). Limited dependent variable models using panel data. J. Human Res..

[36] Owoeye R.S. (2020). Factors influencing cassava farmers' choices of climate adaptation strategies in rainforest agro-ecological zone of southwest, Nigeria. Int. J. Environ. Agric. Res..

[37] Salau E.S., Onuk E.G., Ibrahim A. (2012). Knowledge, perception and adaptation strategies to climate change among farmers in southern agricultural zone of Nasarawa State, Nigeria. J. Agric. Ext..

[38] Oyewole S.O., Sennuga S.O. (2020). Factors influencing sustainable agricultural practices among smallholder farmers in Ogun State of Nigeria. Asian J. Adv. Agric. Res..

[39] Ugwuoke B.C., Attamah C.O. (2019). Linkages between the agricultural development programme and the local government agricultural development in southeast, Nigeria. J. Agric. Ext..

[40] Ravindranath N.H., Sathaye J.A. (2002).

[41] Schlenker W., Lobell D.B. (2010). Robust negative impacts of climate change on African agriculture. Environ. Res. Lett..

[42] Tenge J.D.G., Hella J.P. (2004). Social and economic factors affecting the adoption of soil and water conservation in West Usambara highlands, Tanzania. Land Degrad. Dev..

[43] Hassan R., Nhemachena C. (2008). Determinants of african farmers‟ strategies for adapting to climate change: multinomial choice analysis. Afri. J. . Agric. Res. Econ..

[44] Faleyimu O.I., Agbeja B.O., Olumuyiwa S.A. (2009). Private forestry, the nucleus of forestry development in the southwest Nigeria. Afr. J. Agric. Res. Dev..

